# Supplementary material for: A metrologically traceable protocol for the quantification of trace metals in different types of microplastic
Source: PLoS One. 2020 Jul 20;15(7):e0236120. doi: 10.1371/journal.pone.0236120 (PMC7371195; doi:10.1371/journal.pone.0236120)
Supplement: S1 File — (DOCX) [file pone.0236120.s001.docx]

A metrologically traceable protocol for the quantification of trace metals in different types of microplastic

Lars Hildebrandt^1, 2^, Marcus von der Au^3, 4^, Tristan Zimmermann^1^, Anna Reese^1, 2^, Jannis Ludwig^5^, Daniel Pröfrock^1,*^

*Corresponding author

Daniel Pröfrock (daniel.proefrock@hzg.de, phone +49415287-2846)

^1^ Helmholtz-Zentrum Geesthacht, Institute of Coastal Research, Marine Bioanalytical Chemistry, Max-Planck Str. 1, 21502 Geesthacht, Germany

^2^ Universität Hamburg, Department of Chemistry, Inorganic and Applied Chemistry, Martin-Luther-King-Platz 6, 20146 Hamburg, Germany

^3^ Federal Institute of Hydrology, Department G2 - Aquatic Chemistry, Am Mainzer Tor 1, 56068 Koblenz, Germany

^4^ Federal Institute for Materials Research and Testing, Division 1.1 - Inorganic Trace Analysis, Richard-Willstätter-Str. 11, 12489, Berlin, Germany

^5^ University of Kiel, Department of Chemistry, Institute of Organic Chemistry, Otto-Hahn-Platz 4, 24098 Kiel, Germany

**Table A1**: Typical instrument settings and operating configurations for ICP-MS/MS measurements using the Agilent 8800.

| **Instrument configurations and settings** | | | | |
| --- | --- | --- | --- | --- |
| Sample introduction | Double-pass spray chamber | | | |
| Nebulizer | Self aspirating MicroFlow (ESI) | | | |
| Interface cones | Nickel | | | |
| RF power | 1550 W | | | |
| Carrier gas flow | 1.12 L min^-1^ | | | |
| Make-up gas flow | 0.11 L min^-1^ | | | |
| Used cell gases | He, O_2_, H_2_ | | | |
| **Lens parameters** | **no gas** | **He** | **H_2_** | **O_2_** |
| Extract 1 | -2.5 V | -2.5 V | -2.5 V | -2.5 V |
| Extract 2 | -195.0 V | -195 V | -195 V | -195 V |
| Omega Bias | -115 V | -115 V | -115 V | -115 V |
| Omega Lens | 9.4 V | 9.4 V | 9.4 V | 9.4 V |
| Q1 Entrance | -2 V | -1 V | 0 V | -1 V |
| Q1 Exit | -6 V | -6 V | -3 V | -3 V |
| Cell Focus | 3 V | 1 V | 0 V | 5 V |
| Cell Entrance | -40 V | -50 V | -50 V | -50 V |
| Cell Exit | -50 V | -60 V | -60 V | -60 V |
| Deflect | 14.4 V | -4.2 V | -4 V | 4 V |
| Plate Bias | -50 V | -60 V | -60 V | -60 V |
| **Cell parameters** | **no gas** | **He** | **H_2_** | **O_2_** |
| Cell gas flow | 0 mL/min | 4.5 mL/min | 6 mL/min | 30% |
| OctP Bias | -8 V | -20 V | -18 V | -5 V |
| OctP RF | 130 V | 170 V | 190 V | 180 V |
| Energy Discrimination | 5 V | 5 V | 0 V | -7 V |

**Table A2: Detection modes, isotopes, LOD and LOQ values, and recoveries for the QC standard solution.**

| **Analytes** | | | **Blanks** | |  |  |  |  |
| --- | --- | --- | --- | --- | --- | --- | --- | --- |
| **Mass** | **Element** | **Element + Mode** | ***LOD* [µg/L]** | ***LOQ* [µg/L]** | **Reference *C* [µg/L]** | **Average *C* [µg/L]** | ***SD***  **[µg/L]** | **Recovery [%]** |
| 7 | Li | 7 Li [ No Gas ] | 0.13 | 0.16 | 25 | 23.3 | 1.1 | 93.2 |
| 9 | Be | 9 Be [ No Gas ] | 0.010 | 0.027 | 250 | 265 | 14 | 106.1 |
| 45 | Sc | 45 Sc [ No Gas ] | ≥ 0 | ≥ 0 | 25 | 25.7 | 0.8 | 102.6 |
| 63 | Cu | 63 Cu [ No Gas ] | 0.5 | 0.5 | 25 | 24.9 | 0.6 | 99.7 |
| 66 | Zn | 66 Zn [ No Gas ] | 0.5 | 1.4 | 250 | 261 | 5 | 104.5 |
| 75 | As | 75 As [ No Gas ] | 40 | 40 | 250 | 228 | 12 | 91.3 |
| 82 | Se | 82 Se [ No Gas ] | 1.0 | 1.0 | 250 | 220 | 4 | 87.9 |
| 85 | Rb | 85 Rb [ No Gas ] | 0.021 | 0.021 | 25 | 27.6 | 0.3 | 110.3 |
| 88 | Sr | 88 Sr [ No Gas ] | 0.027 | 0.09 | 25 | 27.9 | 0.3 | 111.7 |
| 89 | Y | 89 Y [ No Gas ] | 0.0013 | 0.004 | 25 | 26.7 | 0.5 | 106.9 |
| 90 | Zr | 90 Zr [ No Gas ] | 0.005 | 0.015 | 0 | 0 | 0 | - |
| 107 | Ag | 107 Ag [ No Gas ] | 0.0019 | 0.0019 | 0 | 0.46 | 0.01 | - |
| 115 | In | 115 In [ No Gas ] | 0.009 | 0.017 | 25 | 26.9 | 0.3 | 107.7 |
| 118 | Sn | 118 Sn [ No Gas ] | 0.11 | 0.11 | 25 | 26.9 | 0.4 | 107.8 |
| 125 | Te | 125 Te [ No Gas ] | 0.008 | 0.021 | 25 | 27.4 | 0.6 | 109.5 |
| 133 | Cs | 133 Cs [ No Gas ] | 0.005 | 0.008 | 0 | 0 | 0 | - |
| 137 | Ba | 137 Ba [ No Gas ] | 0.029 | 0.09 | 25 | 26.7 | 0.4 | 106.9 |
| 139 | La | 139 La [ No Gas ] | 0.0011 | 0.004 | 25 | 27.2 | 0.3 | 108.9 |
| 140 | Ce | 140 Ce [ No Gas ] | 0.003 | 0.011 | 25 | 26.9 | 0.3 | 107.7 |
| 141 | Pr | 141 Pr [ No Gas ] | 0.0003 | 0.0010 | 25 | 27.00 | 0.19 | 108.0 |
| 146 | Nd | 146 Nd [ No Gas ] | 0.0012 | 0.004 | 25 | 26.25 | 0.21 | 105.0 |
| 147 | Sm | 147 Sm [ No Gas ] | 0.0006 | 0.0018 | 25 | 26.20 | 0.20 | 104.8 |
| 153 | Eu | 153 Eu [ No Gas ] | 0.00018 | 0.00018 | 25 | 27.32 | 0.12 | 109.3 |
| 157 | Gd | 157 Gd [ No Gas ] | 0.4 | 0.4 | 25 | 26.22 | 0.17 | 104.9 |
| 159 | Tb | 159 Tb [ No Gas ] | 0.00009 | 0.00029 | 25 | 27.56 | 0.22 | 110.2 |
| 163 | Dy | 163 Dy [ No Gas ] | 0.0003 | 0.0009 | 25 | 26.62 | 0.21 | 106.5 |
| 165 | Ho | 165 Ho [ No Gas ] | ≥ 0 | ≥ 0 | 25 | 27.27 | 0.19 | 109.1 |
| 166 | Er | 166 Er [ No Gas ] | 0.0004 | 0.0013 | 25 | 26.54 | 0.09 | 106.2 |
| 169 | Tm | 169 Tm [ No Gas ] | 0.00023 | 0.0007 | 25 | 27.60 | 0.16 | 110.4 |
| 172 | Yb | 172 Yb [ No Gas ] | 0.0027 | 0.005 | 25 | 26.80 | 0.19 | 107.2 |
| 175 | Lu | 175 Lu [ No Gas ] | ≥ 0 | ≥ 0 | 25 | 27.33 | 0.28 | 109.3 |
| 195 | Pt | 195 Pt [ No Gas ] | 5 | 17 | 0 | 0 | 0 | - |
| 205 | Tl | 205 Tl [ No Gas ] | ≥ 0 | ≥ 0 | 25 | 27.16 | 0.23 | 108.6 |
| 208 | Pb | 208 Pb [ No Gas ] | 0.26 | 0.8 | 25 | 26.44 | 0.23 | 105.7 |
| 209 | Bi | 209 Bi [ No Gas ] | ≥ 0 | ≥ 0 | 25 | 27.67 | 0.22 | 110.7 |
| 232 | Th | 232 Th [ No Gas ] | 0.0004 | 0.0011 | 0 | 0 | 0 | - |
| 238 | U | 238 U [ No Gas ] | 0.00019 | 0.0006 | 25 | 27.1 | 0.3 | 108.5 |
| 23 | Na | 23 Na [ He ] | ≥ 0 | ≥ 0 | 25 | 14 | 7 | 56.2 |
| 39 | K | 39 K [ He ] | ≥ 0 | ≥ 0 | 25 | 11 | 5 | 43.6 |
| 51 | V | 51 V [ He ] | 1.4 | 3 | 25 | 23.5 | 0.5 | 94.1 |
| 52 | Cr | 52 Cr [ He ] | 0.4 | 0.7 | 25 | 24.2 | 0.5 | 96.7 |
| 55 | Mn | 55 Mn [ He ] | 0.05 | 0.08 | 25 | 24.1 | 0.6 | 96.3 |
| 59 | Co | 59 Co [ He ] | 0.007 | 0.017 | 25 | 23.7 | 0.6 | 94.8 |
| 60 | Ni | 60 Ni [ He ] | 0.17 | 0.17 | 25 | 23.7 | 0.6 | 94.8 |
| 63 | Cu | 63 Cu [ He ] | 0.26 | 0.26 | 25 | 23.6 | 0.6 | 94.5 |
| 66 | Zn | 66 Zn [ He ] | 0.4 | 1.2 | 250 | 244 | 5 | 97.5 |
| 71 | Ga | 71 Ga [ He ] | 0.012 | 0.024 | 25 | 24.3 | 0.4 | 97.2 |
| 72 | Ge | 72 Ge [ He ] | 0.4 | 0.4 | 25 | 25.3 | 1.7 | 101.3 |
| 75 | As | 75 As [ He ] | 0.15 | 0.4 | 250 | 235 | 4 | 94.1 |
| 85 | Rb | 85 Rb [ He ] | 0.015 | 0.015 | 25 | 24.9 | 0.4 | 99.7 |
| 88 | Sr | 88 Sr [ He ] | 0.03 | 0.10 | 25 | 25.4 | 0.3 | 101.5 |
| 95 | Mo | 95 Mo [ He ] | 0.004 | 0.010 | 25 | 24.4 | 0.5 | 97.5 |
| 101 | Ru | 101 Ru [ He ] | ≥ 0 | ≥ 0 | 0 | 0 | 0 | - |
| 105 | Pd | 105 Pd [ He ] | 0.0027 | 0.0027 | 0 | 0.09 | 0.01 | - |
| 107 | Ag | 107 Ag [ He ] | 0.0023 | 0.006 | 0 | 0.43 | 0.01 | - |
| 111 | Cd | 111 Cd [ He ] | ≥ 0 | ≥ 0 | 25 | 24.5 | 0.3 | 98.1 |
| 115 | In | 115 In [ He ] | 0.005 | 0.010 | 25 | 24.6 | 0.4 | 98.3 |
| 118 | Sn | 118 Sn [ He ] | 0.08 | 0.08 | 25 | 25.3 | 0.3 | 101.2 |
| 121 | Sb | 121 Sb [ He ] | ≥ 0 | ≥ 0 | 25 | 27.0 | 0.5 | 108.2 |
| 125 | Te | 125 Te [ He ] | ≥ 0 | ≥ 0 | 25 | 25.0 | 1.2 | 100.1 |
| 133 | Cs | 133 Cs [ He ] | 0.004 | 0.005 | 0 | 0 | 0 | - |
| 137 | Ba | 137 Ba [ He ] | 0.025 | 0.08 | 25 | 25.1 | 0.4 | 100.4 |
| 182 | W | 182 W [ He ] | 0.013 | 0.04 | 0 | 0.39 | 0.01 | - |
| 185 | Re | 185 Re [ He ] | 0.00005 | 0.00005 | 0 | 0.36 | 0.02 | - |
| 195 | Pt | 195 Pt [ He ] | 5 | 15 | 0 | 0 | 0 | - |
| 197 | Au | 197 Au [ He ] | 0.014 | 0.018 | 0 | 0.01 | 0.02 | - |
| 205 | Tl | 205 Tl [ He ] | ≥ 0 | ≥ 0 | 25 | 24.89 | 0.25 | 99.5 |
| 206 | Pb | 206 Pb [ He ] | 0.25 | 0.8 | 25 | 24.4 | 0.3 | 97.7 |
| 208 | Pb | 208 Pb [ He ] | 0.27 | 0.9 | 25 | 24.51 | 0.20 | 98.0 |
| 209 | Bi | 209 Bi [ He ] | ≥ 0 | ≥ 0 | 25 | 24.98 | 0.24 | 99.9 |
| 232 | Th | 232 Th [ He ] | 0.0005 | 0.0016 | 0 | 0 | 0 | - |
| 238 | U | 238 U [ He ] | 0.00009 | 0.00029 | 25 | 24.58 | 0.17 | 98.3 |
| 45 | Sc | 45 -> 61 Sc [ O2 ] | ≥ 0 | ≥ 0 | 25 | 23.45 | 0.11 | 93.8 |
| 47 | Ti | 47 -> 63 Ti [ O2 ] | 0.14 | 0.4 | 25 | 24.76 | 0.28 | 99.0 |
| 51 | V | 51 -> 67 V [ O2 ] | 0.06 | 0.06 | 25 | 23.4 | 0.7 | 93.7 |
| 75 | As | 75 -> 91 As [ O2 ] | 0.013 | 0.025 | 250 | 245 | 4 | 98.1 |
| 89 | Y | 89 -> 105 Y [ O2 ] | 0.0006 | 0.0014 | 25 | 24.8 | 0.5 | 99.3 |
| 90 | Zr | 90 -> 106 Zr [ O2 ] | 0.006 | 0.015 | 0 | 0 | 0 | - |
| 95 | Mo | 95 -> 127 Mo [ O2 ] | 0.011 | 0.03 | 25 | 24.3 | 0.4 | 97.2 |
| 115 | In | 115 -> 115 In [ O2 ] | 0.007 | 0.014 | 25 | 25.1 | 0.4 | 100.3 |
| 118 | Sn | 118 -> 118 Sn [ O2 ] | 0.10 | 0.10 | 25 | 25.1 | 0.4 | 100.4 |
| 139 | La | 139 -> 155 La [ O2 ] | 0.0016 | 0.005 | 25 | 25.28 | 0.25 | 101.1 |
| 140 | Ce | 140 -> 156 Ce [ O2 ] | 0.0021 | 0.007 | 25 | 25.41 | 0.24 | 101.6 |
| 141 | Pr | 141 -> 157 Pr [ O2 ] | 0.0005 | 0.0017 | 25 | 25.29 | 0.26 | 101.2 |
| 146 | Nd | 146 -> 162 Nd [ O2 ] | 0.0005 | 0.0016 | 25 | 24.43 | 0.25 | 97.7 |
| 147 | Sm | 147 -> 163 Sm [ O2 ] | 0.0007 | 0.0023 | 25 | 24.5 | 0.3 | 98.1 |
| 153 | Eu | 153 -> 169 Eu [ O2 ] | ≥ 0 | ≥ 0 | 25 | 24.44 | 0.29 | 97.7 |
| 157 | Gd | 157 -> 173 Gd [ O2 ] | 0.4 | 0.5 | 25 | 24.65 | 0.22 | 98.6 |
| 159 | Tb | 159 -> 175 Tb [ O2 ] | 0.00020 | 0.0006 | 25 | 25.28 | 0.17 | 101.1 |
| 163 | Dy | 163 -> 179 Dy [ O2 ] | ≥ 0 | ≥ 0 | 25 | 24.30 | 0.27 | 97.2 |
| 165 | Ho | 165 -> 181 Ho [ O2 ] | 0.00018 | 0.0006 | 25 | 25.35 | 0.20 | 101.4 |
| 166 | Er | 166 -> 182 Er [ O2 ] | ≥ 0 | ≥ 0 | 25 | 24.7 | 0.3 | 98.9 |
| 169 | Tm | 169 -> 185 Tm [ O2 ] | 0.00011 | 0.0004 | 25 | 24.97 | 0.25 | 99.9 |
| 172 | Yb | 172 -> 188 Yb [ O2 ] | 0.0024 | 0.008 | 25 | 24.59 | 0.24 | 98.4 |
| 175 | Lu | 175 -> 191 Lu [ O2 ] | 0.00005 | 0.00018 | 25 | 25.18 | 0.24 | 100.7 |
| 182 | W | 182 -> 214 W [ O2 ] | 0.005 | 0.012 | 0 | 0.01 | 0.01 | - |
| 185 | Re | 185 -> 217 Re [ O2 ] | ≥ 0 | ≥ 0 | 0 | 0 | 0 | - |
| 7 | Li | 7 -> 7 Li [ H2 ] | 0.5 | 1.2 | 25 | 24.5 | 0.9 | 97.9 |
| 24 | Mg | 24 -> 24 Mg [ H2 ] | 1.1 | 3.0 | 25 | 24.3 | 0.5 | 97.3 |
| 27 | Al | 27 -> 27 Al [ H2 ] | 3 | 10 | 25 | 25.0 | 0.5 | 100.1 |
| 39 | K | 39 -> 39 K [ H2 ] | 13 | 25 | 25 | 23.8 | 0.9 | 95.1 |
| 40 | Ca | 40 -> 40 Ca [ H2 ] | 2.9 | 8 | 2500 | 2350 | 80 | 93.9 |
| 47 | Ti | 47 -> 47 Ti [ H2 ] | 0.3 | 1.0 | 25 | 25.6 | 0.9 | 102.2 |
| 52 | Cr | 52 -> 52 Cr [ H2 ] | 4 | 10 | 25 | 24.9 | 0.7 | 99.4 |
| 55 | Mn | 55 -> 55 Mn [ H2 ] | 0.07 | 0.14 | 25 | 24.7 | 0.5 | 99.0 |
| 56 | Fe | 56 -> 56 Fe [ H2 ] | 1.2 | 4 | 250 | 235 | 5 | 94.1 |
| 59 | Co | 59 -> 59 Co [ H2 ] | 0.021 | 0.04 | 25 | 24.6 | 0.4 | 98.4 |
| 60 | Ni | 60 -> 60 Ni [ H2 ] | 0.3 | 0.3 | 25 | 24.3 | 0.6 | 97.0 |
| 69 | Ga | 69 -> 69 Ga [ H2 ] | 0.021 | 0.05 | 25 | 25.2 | 0.5 | 100.6 |
| 78 | Se | 78 -> 78 Se [ H2 ] | 0.030 | 0.05 | 250 | 247 | 4 | 98.6 |
| 111 | Cd | 111 -> 111 Cd [ H2 ] | 0.0003 | 0.0003 | 25 | 24.7 | 0.4 | 98.9 |
| 115 | In | 115 -> 115 In [ H2 ] | 0.007 | 0.0140 | 25 | 25.1 | 0.4 | 100.4 |
| 118 | Sn | 118 -> 118 Sn [ H2 ] | 0.0003 | 0.0003 | 25 | 25.7 | 0.3 | 103.0 |
| 137 | Ba | 137 -> 137 Ba [ H2 ] | 0.03 | 0.11 | 25 | 25.4 | 0.4 | 101.4 |
| 200 | Hg | 200 -> 200 Hg [ H2 ] | ≥ 0 | ≥ 0 | 25 | 1.1 | 1.0 | 4.5 |
| 201 | Hg | 201 -> 201 Hg [ H2 ] | ≥ 0 | ≥ 0 | 25 | 1.1 | 1.0 | 4.3 |
| 202 | Hg | 202 -> 202 Hg [ H2 ] | ≥ 0 | ≥ 0 | 25 | 1.2 | 1.1 | 4.7 |

**Table A3: Uncertainty calculation for the Cd content of ERM^®^-EC680m and BAM-H010 applying the *Kragten* spreadsheet approach (custom written in Excel^©^).**

**Table A4: Uncertainty calculation for the Pb content of ERM^®^-EC680m and BAM-H010 applying the *Kragten* spreadsheet approach (custom written in Excel^©^).**

**Table A5-A11: Concentrations of heavy metals in the six investigated plastic CRMs.**

| **ERM^®^-EC680m** | | | | | |
| --- | --- | --- | --- | --- | --- |
| **Metal** | **Certified** | | **ICP-MS/MS results** | | |
|  | **Mass fraction [µg/kg]** | ***U* (*k* = 2)**  **[µg/kg]** | **Mass fraction [µg/kg]** | ***SD* [µg/kg]** | **Recovery [%]** |
| **As** | 4700 | 400 | 4730 | 170 | 101 |
| **Cd** | 20800 | 900 | 21700 | 700 | 104 |
| **Cr** | 9600 | 50 | 9600 | 400 | 100 |
| **Hg** | 2560 | 160 | 2670 | 150 | 105 |
| **Pb** | 11300 | 400 | 11600 | 600 | 103 |
| **Sn** | 9600 | 700 | 10000 | 500 | 104 |
| **Sb** | 20700 | 1600 | 21000 | 1000 | 104 |
| **Zn** | 194000 | 12000 | 205000 | 7000 | 106 |

| **ERM^®^-EC681m** | | | | | |
| --- | --- | --- | --- | --- | --- |
| **Metal** | **Certified** | | **ICP-MS/MS results** | | |
|  | **Mass fraction [µg/kg]** | ***U (k* = 2) [µg/kg]** | **Mass fraction [µg/kg]** | ***SD* [µg/kg]** | **Recovery [%]** |
| **As** | 17000 | 1200 | 16800 | 500 | 99 |
| **Cd** | 146000 | 5000 | 151000 | 4000 | 103 |
| **Cr** | 45100 | 1900 | 44600 | 700 | 99 |
| **Hg** | 9900 | 800 | 10100 | 400 | 102 |
| **Pb** | 69700 | 2500 | 71000 | 3000 | 101 |
| **Sn** | 86000 | 7000 | 90000 | 1800 | 104 |
| **Sb** | 99000 | 6000 | 102500 | 2800 | 103 |
| **Zn** | 1170000 | 40000 | 1210000 | 40000 | 103 |

| **NMIJ 8123-a** | | | | | |
| --- | --- | --- | --- | --- | --- |
| **Metal** | **Certified** | | **ICP-MS/MS results** | | |
|  | **Mass fraction [µg/kg]** | ***U (k* = 2) [µg/kg]** | **Mass fraction [µg/kg]** | ***SD* [µg/kg]** | **Recovery [%]** |
| **As** | - | - | 21 | 15 | - |
| **Cd** | 95620 | 1390 | 98000 | 3000 | 102 |
| **Cr** | 949000 | 9700 | 950000 | 40000 | 100 |
| **Hg** | 937000 | 19400 | 102000 | 17000 | 108 |
| **Pb** | 965500 | 6600 | 972000 | 23000 | 101 |
| **Sn** | - | - | - | - | - |
| **Sb** | - | - | 23 | 10 | - |
| **Zn** | - | - | 510000 | 70000 | - |

| **NMIJ 8133-a** | | | | | |
| --- | --- | --- | --- | --- | --- |
| **Metal** | **Certified** | | **ICP-MS/MS results** | | |
|  | **Mass fraction [µg/kg]** | ***U (k* = 2) [µg/kg]** | **Mass fraction [µg/kg]]** | ***SD* [µg/kg]** | **Recovery [%]** |
| **As** | - | - | 11 | 8 | - |
| **Cd** | 94260 | 1390 | 96000 | 3000 | 102 |
| **Cr** | 895200 | 9600 | 929000 | 13000 | 104 |
| **Hg** | 941500 | 19600 | 1055000 | 29000 | 112 |
| **Pb** | 949200 | 7500 | 1000000 | 40000 | 105 |
| **Sn** | - | - | - | - | - |
| **Sb** | - | - | - | - | - |
| **Zn** | - | - | 112000 | 5000 | - |

| **BAM-H010** | | | | | |
| --- | --- | --- | --- | --- | --- |
| **Metal** | **Certified** | | **ICP-MS/MS results** | | |
|  | **Mass fraction [µg/kg]** | ***U (k* = 2) [µg/kg]** | **Mass fraction [µg/kg]** | ***SD* [µg/kg]** | **Recovery [%]** |
| **As** | - | - | 26 | 19 | - |
| **Cd** | 93000 | 5000 | 103000 | 2500 | 111 |
| **Cr** | 470000 | 36000 | 1400 | 110 | 0.30 |
| **Hg** | 415000 | 27000 | 404000 | 8000 | 97 |
| **Pb** | 479000 | 17000 | 524000 | 14000 | 109 |
| **Sn** | - | - | - | - | - |
| **Sb** | - | - | - | - | - |
| **Zn** | - | - | 179 | 70 | - |

| **Lead in Plastic - QC** | | | | | |
| --- | --- | --- | --- | --- | --- |
| **Metal** | **Certified** | | **ICP-MS/MS results** | | |
|  | **Mass fraction [µg/kg]** | ***U (k* = 2) [µg/kg]** | **Mass fraction [µg/kg]** | ***SD* [µg/kg]** | **Recovery [%]** |
| **As** | - | - | 137 | 12 | - |
| **Cd** | - | - | 43 | 4 | - |
| **Cr** | - | - | 22000 | 3000 | - |
| **Hg** | - | - | 600 | 1000 | - |
| **Pb** | 376000 | 18900 | 360600 | 1500 | 96 |
| **Sn** | - | - | 64 | 17 | - |
| **Sb** | - | - | 4200 | 600 | - |
| **Zn** | - | - | 15800 | 1900 | - |

**Table A12: Calculated combined uncertainties for the certified metals contained in ERM^®^-EC680m and BAM-H010.**

| **Metal** | **ERM^®^-EC680m** | | **BAM-H010** | |
| --- | --- | --- | --- | --- |
|  | ***U* (*k*=2) [µg kg^-1^])** | ***U*_rel_ (%)** | ***U* (*k*=2) [µg kg^-1^])** | ***U*_rel_ (%)** |
| As | 700 | 8 | - | - |
| Cd | 1700 | 5 | 5000 | 2.4 |
| Cr | 800 | 6 | 230 | 9 |
| Hg | 400 | 6 | 19000 | 2.3 |
| Pb | 1200 | 6 | 28000 | 2.7 |
| Sn | 900 | 5 | - | - |
| Sb | 2000 | 5 | - | - |
| Zn | 16000 | 6 | - | - |

**Table A13: Percentages of the different errors contributing to the combined uncertainties.**

| **Metal** | **ERM^®^-EC680m** | | | | **BAM-H010** | | | |
| --- | --- | --- | --- | --- | --- | --- | --- | --- |
|  | ***Δß*_measurement precision_** | ***ΔV*_dilution_** | ***Δm*_CRM_** | ***ΔC*_digest replicates_** | ***Δß*_measurement precision_** | ***ΔV*_dilution_** | ***Δm*_CRM_** | ***ΔC*_digest replicates_** |
| **As** | 57% | 0.07% | 0.005% | 43% | - | - | - | - |
| **Cd** | 12% | 0.16% | 0.012% | 88% | 7% | 0.7% | 0.05% | 93% |
| **Cr** | 14% | 0.13% | 0.009% | 86% | 7% | 0.05% | 0.003% | 93% |
| **Hg** | 9% | 0.12% | 0.008% | 91% | 40% | 0.8% | 0.05% | 60% |
| **Pb** | 9% | 0.13% | 0.009% | 91% | 5% | 0.6% | 0.04% | 94% |
| **Sn** | 3% | 0.15% | 0.011% | 97% | - | - | - | - |
| **Sb** | 8% | 0.16% | 0.011% | 92% | - | - | - | - |
| **Zn** | 9% | 0.14% | 0.010% | 91% | - | - | - | - |

**
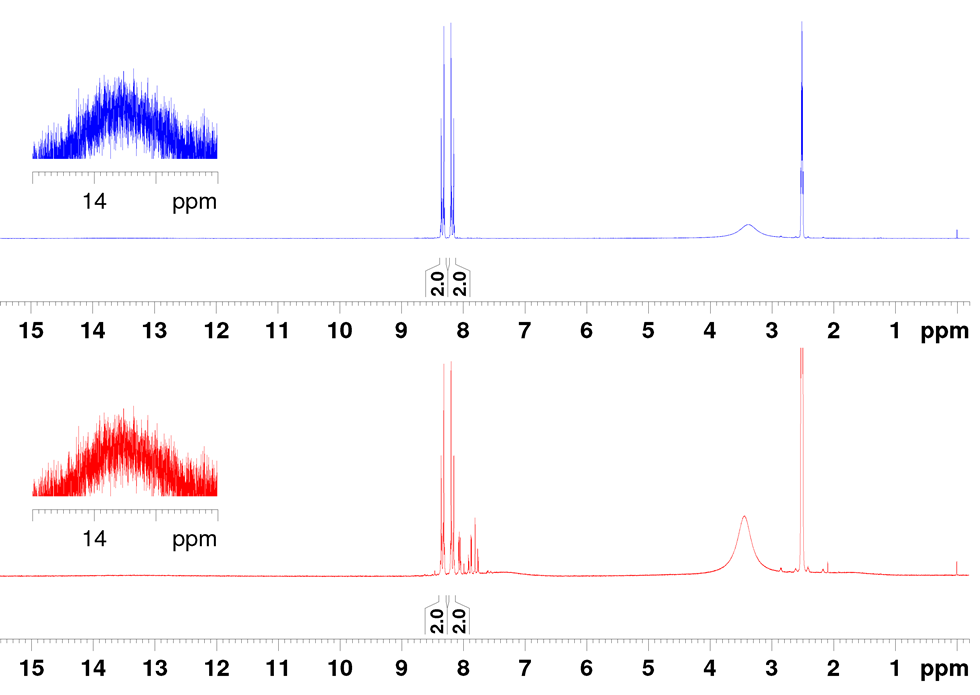
**

**Figure A1: ^1^H NMR (200 MHz, 300 K, DMSO-d_6_, TMS) spectra of commercial 4-nitrobenzoic acid from Merck (blue spectrum) and of the yellowish precipitate obtained after acid digestion of BAM-H010 (red spectrum) with enlarged section of the acidic proton.**

**^1^H NMR (200 MHz, DMSO-d_6_, 300 K, TMS): δ = 13.30 (s, 1H, COOH), 8.39-8.29 (m, 2H, NCCH), 8.23-8.13 (m, 2H, CCCH) ppm.**
